# Synthesis Chalones and Their Isomerization into Flavanones and Azaflavanones

**DOI:** 10.3390/mps2030070

**Published:** 2019-08-15

**Authors:** Djenisa H. A. Rocha, Patrícia A. A. M. Vaz, Diana C. G. A. Pinto, Artur M. S. Silva

**Affiliations:** QOPNA & LAQV-REQUIMTE, Department of Chemistry, University of Aveiro, Campus de Santiago, 3810-193 Aveiro, Portugal

**Keywords:** flavanones, 2-aryl-2,3-dihydroquinolin-4(1*H*)-ones, chalcones, microwaves, proton sponge, montmorillonite K10

## Abstract

Flavanones [2-aryl-2,3-dihydrochromen-4(1*H*)ones] and 2-aryl-2,3-dihydroquinolin-4(1*H*)-ones are valuable precursors in the synthesis of important pharmacological scaffolds, so efficient methodologies towards their synthesis are important in the medicinal chemistry context. Their synthesis also involves theoretical concepts such as aldol condensation, isomerization, and catalysis that make it useful in an undergraduate organic chemistry laboratory. The use of both microwave irradiation as a source of energy to promote reactions and efficient catalysts are considered within green chemistry principles, mostly because the reaction yields are improved and reaction time decreased. In this paper, the efficiency of microwave irradiation use in the synthesis of chalcone derivatives and efficient catalyst systems to promote their isomerization into flavanones and 2-aryl-2,3-dihydroquinolin-4(1*H*)-ones is demonstrated.

## 1. Introduction

Chalcones are naturally occurring compounds, more recognized as the precursors of other flavonoids, but both natural and synthetic derivatives present interesting biological activities [1]. Furthermore, their synthesis is also investigated due to their use in the synthesis of other biologically important compounds, the flavanones and 2-aryl-2,3-dihydroquinolin-4(1*H*)-ones. Flavanones are also naturally occurring compounds belonging to the flavonoid family and are valuable precursors in the synthesis of flavones. Both are important secondary metabolites and their Human health-promoting effects are well documented [2,3]. On the other hand, 2-aryl-2,3-dihydroquinolin-4(1*H*)-ones are valuable precursors in the synthesis of quinolin-4(1*H*)-ones, which are recognized for their use in medicine [4].

**Scheme 1:**
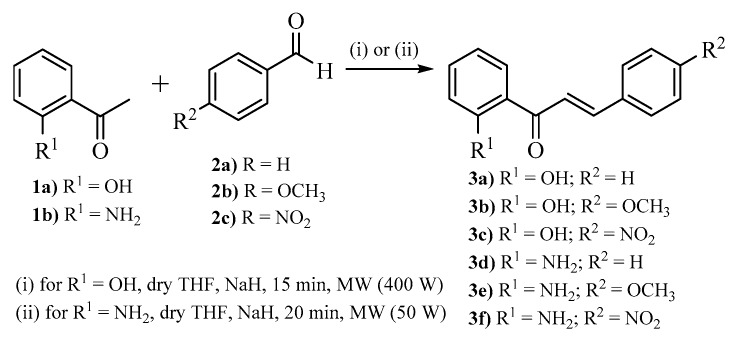
Synthesis of chalcone derivatives.

**Scheme 2:**
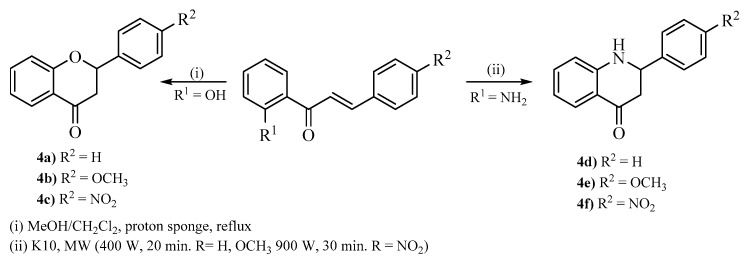
Synthesis of flavanone and 2-aryl-2,3-dihydroquinolin-4(1*H*)-one derivatives.

Considering the significance of flavanones and 2-aryl-2,3-dihydroquinolin-4(1*H*)-ones, their synthesis is still investigated by organic researchers, and nowadays, greener methodologies are being searched for [5,6,7,8,9]. Despite the several methodologies reported, the most common starting material used in their synthesis are chalcones 3 with the appropriate substitution pattern, a 2’-hydroxyl group for flavanones 4a–c, and a 2’-amino group for 2-aryl-2,3-dihydroquinolin-4(1H)-ones 4d–f (Scheme 1 and Scheme 2). Although the main topic of this protocol is the isomerization step, the synthesis of chalcone derivatives will also be presented due to the interesting use of microwave irradiation in their synthesis, as well the theoretical organic concepts involved.

Another important aspect in the two-step methodology herein presented and discussed is its effectiveness with both electron-donating (OCH_3_) and electron-withdrawn (NO_2_) groups, giving good yields in all cases, a fact that is not at all common. Actually, most of the protocols do not include both groups. The approaches described hereafter are operationally very simple and will allow their use in the study of organic synthesis concepts such as aldol condensation and isomerization [10].

## 2. Experimental Design

One of the most common and efficient methodologies towards the synthesis of chalcones is the aldol condensation between acetophenones and benzaldehydes (Scheme 1). The yields are usually very good and the reaction occurs at room temperature. Depending on the substitution pattern and the solvent/base system used, the reaction time varies from 3 to 20 h [1,11,12]. The system of methanol/NaOH is not suitable for the synthesis of nitro-derivatives nor for the synthesis of 2’-aminochalcones. In these cases, the use of a system involving dried tetrahydrofuran (THF)/NaH allowed the synthesis of all types of derivatives in very good yields (above 70%); however, in some cases, it is with long reaction times. Thus, a protocol using microwave irradiation is presented herein and its advantages are the increase of yield, the shortening of the reaction time, and its applicability to the synthesis of both 2’-hydroxychalcones 3a–c and 2’-aminochalcones 3d–f (Scheme 1).

The isomerization of chalcones into flavanones is usually performed using inorganic acids and requires careful purification. Herein, we describe a new protocol with easier purification steps using a proton sponge, a tertiary amine capable of catalyzing the isomerization of 2’-hydroxychalcones into the flavanone derivatives 4a–c in good yield (Scheme 2). On the other hand, the isomerization of 2’-aminochalcones was efficiently performed in montmorillonite K10, and, using microwave irradiation, the desired 2-aryl-2,3-dihydroquinolin-4(1*H*)-ones 4d–f (Scheme 2) were obtained in good yields.

### 2.1. Materials

- Acetophenones (2’-aminoacetophenone and 2’-hydroxyacetophnone from Aldrich, Saint Louis, MO, USA).
- Benzaldehydes (e.g., benzaldehyde and *p*-methoxybenzaldehyde from Merck, Kenilworth, NJ, USA; and *p*-nitrobenzaldehyde from Riedel-Haën, Seelze, Germany).
- Sodium hydride from Aldrich (dry, 90%, or 60% dispersion in mineral oil).
- Montmorillonite K10 from Fulka AG.
- Proton sponge from Aldrich.
- Solvents (THF, methanol, ethanol, hexane, ethyl acetate, and dichloromethane p.a. grade from Riedel-Haën).
- Solid supports (silica gel 60 and celite from Merck).

### 2.2. Equipment

- Stirring plate and water circulating system.
- UV lamp (λ = 254 nm).
- Ethos MicroSynth Labstation microwave (Milestone Inc., Sorisole, Italy).
- NMR spectrometer (Bruker Avance 300).

## 3. Procedure

In the following sections, the better procedures will be described in detail. Additional safety recommendations will be included to help the execution of these syntheses in laboratory classrooms.

### 3.1. Synthesis of Chalcone Derivatives (Time for Completion: 45 min)

**Figure 1:**
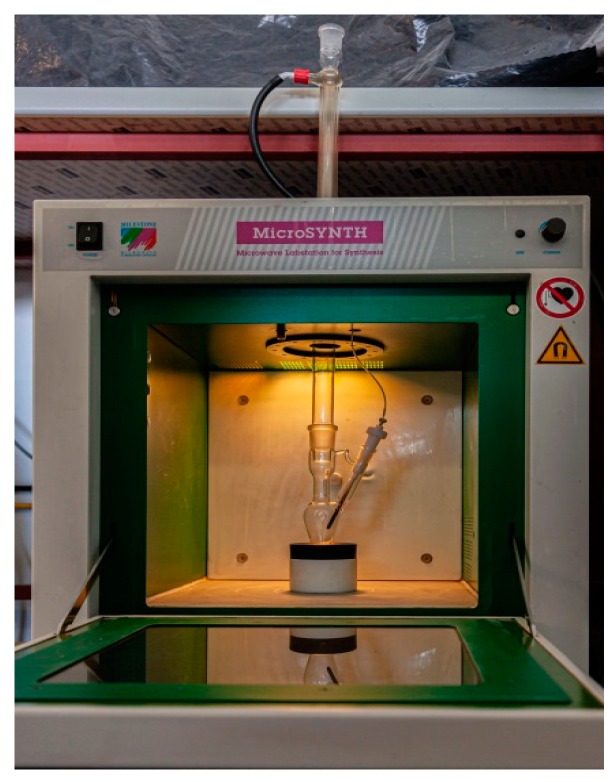
Experimental apparatus for microwave-assisted synthesis of chalcone derivatives.

1. Add 2’-hydroxyacetophenone 1a or 2’-aminoacetophenone 1b (8.2 mmol), dried THF (20.0 mL), and NaH (24.7 mmol) to a round-bottom reactor equipped with a stir bar.
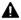
**CRITICAL STEP.** The NaH should be added slowly (if necessary, a glass funnel can be used) and with gentle stirring. The round-bottom reactor can be put in an ice bath placed on a stirring plate. This will prevent the uncontrolled release of hydrogen.
2. **OPTIONAL STEP.** The THF can be dried by the students following a described procedure [13] or molecular sieves can be added to the flask the day before the class.
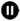
**PAUSE STEP.** After the addition of the NaH, the mixture should be stirred at room temperature for 10 min.
3. Add 1.5 molar equiv. of the desired benzaldehyde 2a–c and place the reactor in the microwave accordingly to the apparatus shown in Figure 1.
4. Maintain the reaction mixture, stirring 15 min and with 400 W irradiation (for 2’-hydroxychalcones 3a–c) or 20 min and with 50 W irradiation (for 2’-aminochalcones 3d–f).
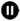
**PAUSE STEP.** After stopping, allow the reaction mixture to cool down to room temperature.
5. Pour the reaction mixture into a vessel containing ice and add hydrochloric acid (1 mol dm^−3^ solution) to adjust pH to below 4 and simultaneously precipitate the desired chalcones.
6. Filter the formed solid using a vacuum filtration apparatus.
7. **OPTIONAL STEPS.** Recrystallize from hot ethanol (1 g of product in 5 mL of hot ethanol or dissolve with dichloromethane and purify by chromatography (using a mixture of hexane/ethyl acetate [7:3]).

### 3.2. Synthesis of Flavanone Derivatives (Time for Completion: 24 h)

**Figure 2:**
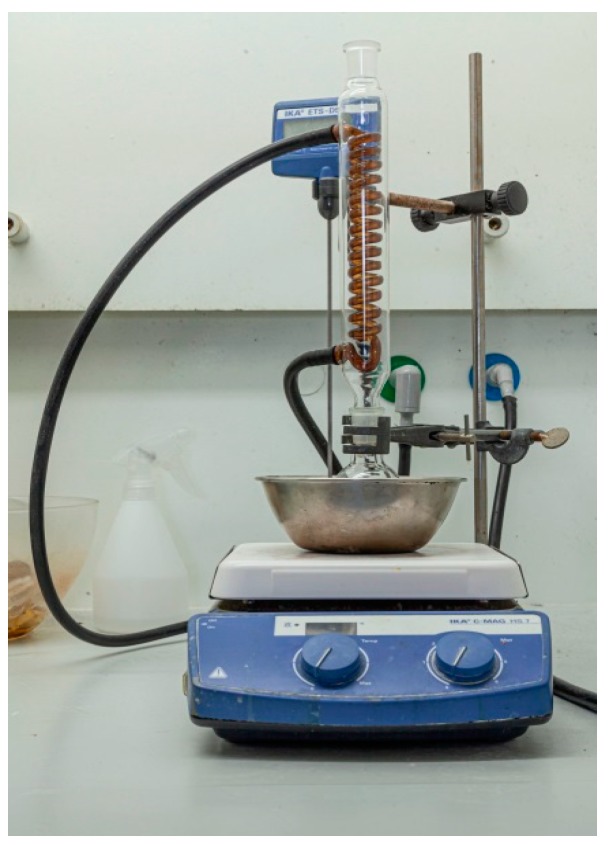
Experimental apparatus for the synthesis of flavanones.

1. Add the appropriate 2’-hydroxychalcones 3a–c (0.2 mmol), methanol (3.0 mL), dichloromethane (3.0 mL), and proton sponge (0.5 mmol) to a 10 mL round-bottom flask equipped with a stir bar.
2. Reflux during 24 h (Figure 2).
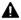
**CRITICAL STEP.** The 2’-hydroxychalcone isomerization is slow, so the reaction progress is controlled by TCL (using a mixture of hexane/dichloromethane [3:7]), and after 12 h, more or less half of the 2’-hydroxychalcone is converted. The reaction can be stopped if an additional step of purification is important for students.
3. Pour the reaction mixture into a vessel containing ice and add hydrochloric acid to adjust pH (near 6).
4. Extract with dichloromethane and evaporate to dryness.
5. **OPTIONAL STEP.** Dissolve with dichloromethane and purify by chromatography (using a mixture of hexane/dichloromethane [3:7]).
6. Recrystallize from hot ethanol (1 g of product in 5 mL of hot ethanol).

### 3.3. Synthesis of 2-aryl-2,3-dihydroquinolin-4(1H)-one Derivatives (Time for Completion: 45 min)

**Figure 3:**
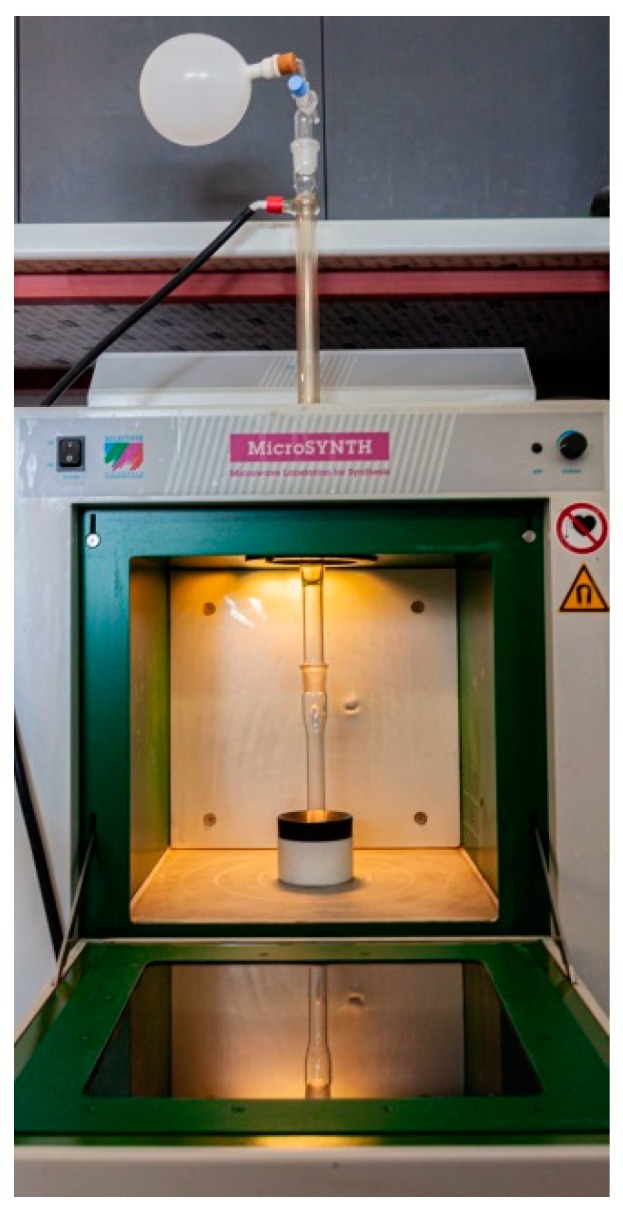
Experimental apparatus for 2-aryl-2,3-dihydroquinolin-4(1*H*)-ones microwave-assisted synthesis.

1. Add 2’-aminochalcone 3d–f (0.1 g), and montmorillonite K10 (1.0 g) to a microwave tube.
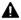
**CRITICAL STEP.** The mixture of the reagents should be homogeneous, so it can be mixed using a mortar and pestle prior.
2. Place the microwave tube accordingly into the apparatus shown in Figure 3 and irradiate at a constant power of 400 W for 20 min for the derivatives 4e,f and 900 W for 30min for the derivative 4d.
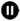
**PAUSE STEP.** After stopping, allow the reaction mixture to cool down to room temperature.
3. Filter the reaction mixture through celite, using ethyl acetate to remove the desired compound.
4. Evaporate the solvent.
5. **OPTIONAL STEP.** Recrystallize from hot ethanol.

## 4. Expected Results

### 4.1. Reaction Conditions

The protocols detailed above are the result of some studies where, among others, reaction times and sources of energy were carefully varied. The chalcones are obtained even if the protocol conditions were not totally followed. The yields are, however, lower and the desired chalcones may need extra purifications steps. Using the best fitted conditions the expected yields are the ones reported in Table 1.

The crucial aspect in the synthesis of flavanone derivatives is the reaction time. It is possible to have flavanones after a few hours but with less than 20% yield, and longer reaction times also decrease the yields to 50%. The best yields are also reported in Table 1.

Finally, in the synthesis of 2-aryl-2,3-dihydroquinolin-4(1*H*)-one derivatives, it is important to perform a homogeneous mixture with the solid support; the use of a mortar and pestle is highly recommended. With less homogeneous mixtures, not only are the yields lower, but, in some cases, the isomerization also does not occur.

It is interesting to analyze the yields (Table 1) and detect that the presence of the strong withdrawing-group decrease the yields. This can be used to encourage a discussion about the mechanisms involved and the nitro group influence.

### 4.2. Product Characterization

**Figure 4:**
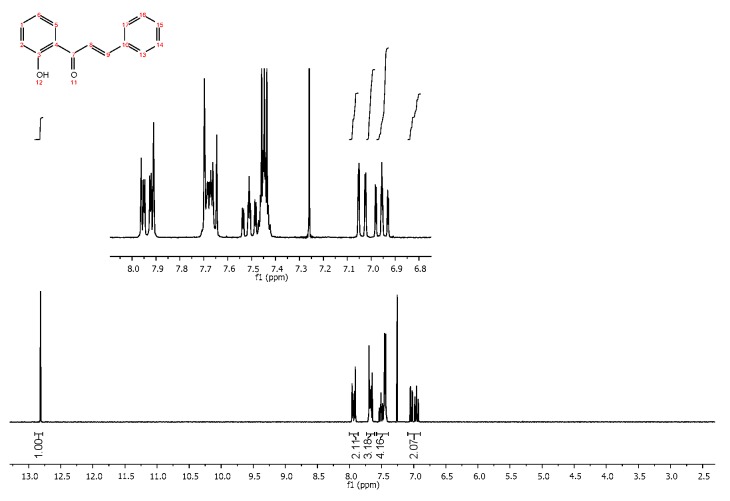
^1^H NMR spectrum of the 2’-hydroxychalcone 3a.

**Figure 5:**
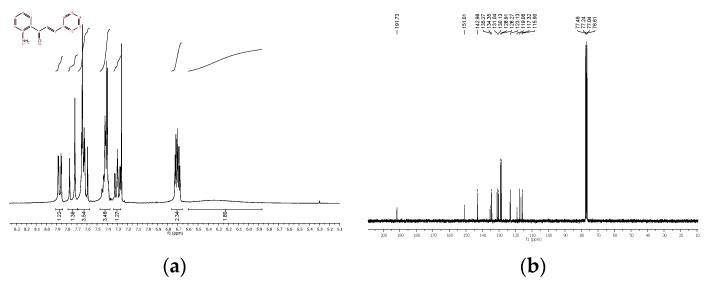
(**a**) ^1^H NMR spectrum of the 2’-aminochalcone 3d; (**b**) ^13^C NMR spectrum of the 2’-aminochalcone 3d.

All compounds can be characterized by NMR spectroscopy and the spectra can be used as examples to teach important NMR features. For example, from the NMR of chalcone derivatives, one can highlight the vinylic system, the deshielded singlet due to the hydroxyl proton that is involved in an intramolecular hydrogen bond (Figure 4). In the case of the 2’-aminochalcones, the detection of these amino protons is more difficult (Figure 5a). In the carbon-13 spectra of chalcone derivatives, the most important signal is the more deshielded one (Figure 5b) due to the carbonyl carbon (~δ 192 ppm).

**Figure 6:**
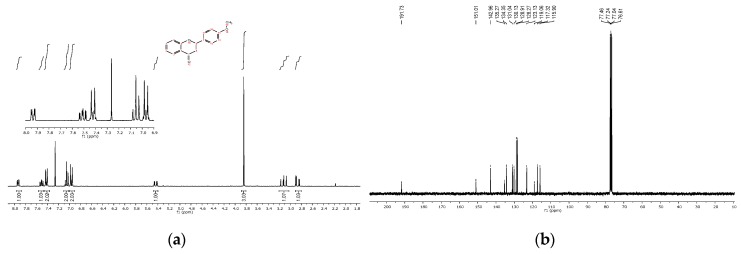
(**a**) ^1^H NMR spectrum of the 4-methoxyflavanone 4b; (**b**) ^13^C NMR spectrum of the 4-methoxyflavanone 4b.

From the ^1^H NMR spectra of flavanone derivatives, the three less shielded signals due to protons H-2 and H-3 should be emphasized. In fact, the H-3 protons are nonequivalent due to the chirality of the carbon C-2, and they appear as two sets of doublet of doublets around δ 3 ppm. Naturally, the H-2 signal multiplicity is also affected by the nonequivalence of the H-3 protons (Figure 6a). The ^13^C NMR spectra of this type of compound presents two signals characteristic of aliphatic carbons—and also the carbonyl carbon signal with similar chemical shift—when compared with its chalcone isomer (Figure 6b).

**Figure 7:**
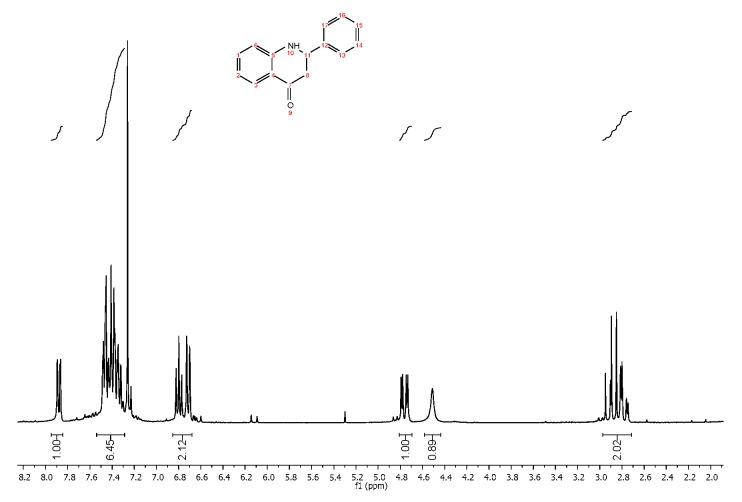
^1^H NMR spectrum of 2-aryl-2,3-dihydroquinolin-4(1*H*)-one 4d.

The only aspect that is different in the case of 2-aryl-2,3-dihydroquinolin-4(1*H*)-one derivatives is the broad singlet at ~δ 4.5 ppm due to the NH proton (Figure 7).

The procedures described proved to be efficient due to the good yields of the desired compounds. The most significant examples were presented, but the methodology can be applied to other substituents, such as halogen and alkyl groups [14,15]. Moreover, the use of microwave irradiation and efficient catalysts to promote the reactions are considered within green chemistry principles.

## Figures and Tables

**Table 1:** Expected yields under optimal conditions.

Compound	R ^1^	R ^2^	Yield * (%)
3a	OH	H	84
3b	OH	OCH_3_	88
3c	OH	NO_2_	80
3d	NH_2_	H	87
3e	NH_2_	OCH_3_	83
3f	NH_2_	NO_2_	78
4a	OH	H	70
4b	OH	OCH_3_	74
4c	OH	NO_2_	69
4d	NH	H	80
4e	NH	OCH_3_	84
4f	NH	NO_2_	60

* Average of four experiments.

## References

[1] Rosa G.P., Seca A.M.L., Barreto M.C., Silva A.M.S., Pinto D.C.G.A. (2019). Chalcones and flavanones bearing hydroxyl and/or methoxyl groups: Synthesis and biological assessments. App. Sci..

[2] Tomás-Navarro M., Vallejo F., Tomás-Barberán F.A. (2014). Bioavailability and metabolism of citrus fruit beverage flavanones in Humans. Polyphenols in Human Health and Disease.

[3] Murkovic M. (2003). Phenolic compounds. Encyclopedia of Food Sciences and Nutrition.

[4] European Medicines Agency EMA/175398/2019. https://www.ema.europa.eu/en/documents/referral/quinolone-fluoroquinolone-article-31-referral-disabling-potentially-permanent-side-effects-lead_en.pdf.

[5] Shinde B., Kamble S.B., Pore D.M., Gosavi P., Gaikwad A., Jadhav H.S., Karale B.K., Burungale A.S. (2018). pH-Transformed ZnO-NPs/NaPTS: The first room-temperature brisk synthesis of flavanones in aqueous medium. Chem. Select.

[6] Gupta A., Jamatia R., Patil R.A., Ma Y.R., Pal A.K. (2018). Copper oxide/reduced graphene oxide nanocomposite-catalyzed synthesis of flavanones and flavanones with triazole hybrid molecules in one pot: A green and sustainable approach. ACS Omega.

[7] Chelghoum M., Bahnous M., Bouraiou A., Bouacida S., Belfaitah A. (2012). An efficient and rapid intramolecular aza-Michael addition of 2’-aminochalcones using ionic liquids as recyclable reaction media. Tetrahedron Lett..

[8] Cheng S., Zhao L., Yu S. (2014). Enantioselective synthesis of azaflavanones using organocatalytic 6-*endo* aza-Michael addition. Adv. Synth. Catal..

[9] Sakirolla R., Yaeghoobi M., Rahman N.A. (2012). Synthesis of flavanones, azaflavanones, and thioflavanones catalyzed by PMA-SiO_2_ as a mild, efficient, and reusable catalyst. Monastsh. Chem..

[10] McMurry J. (2016). Organic Chemistry.

[11] Silva A.M.S., Tavares H.R., Barros A.I.N.R.A., Cavaleiro J.A.S. (1997). NMR and structural and conformational features of 2′-hydroxychalcones and flavones. Spectrosc. Lett..

[12] Barros A.I.N.R.A., Silva A.M.S. (2006). Efficient synthesis of nitroflavones by cyclodehydrogenation of 2’-hydroxychalcones and by Baker-Venkataraman method. Monatsh. Chem..

[13] Furniss B.S., Hannaford A.J., Smith P.W.G., Tatchell A.R. (1989). Vogel’s Textbook of Practical Organic Chemistry.

[14] Silva A.M.S. (1993). Synthesis and Structural Characterization of Flavonoids and Related Compounds. Ph.D. Thesis.

[15] Rocha D.H.A. (2015). Synthesis and Transformation of Chromones and 4-Quinolones. Ph.D. Thesis.

